# Electroencephalographic neurofeedback training can decrease conscious motor control and increase single and dual-task psychomotor performance

**DOI:** 10.1007/s00221-020-05935-3

**Published:** 2020-11-09

**Authors:** Amanpreet Sidhu, Andrew Cooke

**Affiliations:** 1 School of Sport, Health and Exercise Sciences, Bangor University, George Building, Gwynedd, Bangor, LL57 2PZ UK; 2 Te Huataki Waiora School of Health, University of Waikato, Hamilton, New Zealand

**Keywords:** Brain-training, Timed-up-and-go, Automaticity, Motor control, Human movement, Reinvestment

## Abstract

The control of human movements is thought to automize with repetition, promoting consistent execution and reduced dual-task costs. However, contingencies such as illness or constraints to regular movement patterns can promote conscious motor control, which can reduce movement proficiency and make dual-task situations more difficult. This experiment evaluated whether electroencephalographic neurofeedback training can reduce the adverse effects of conscious motor control. Twenty-five participants completed the timed-up-and-go task while wearing a leg brace to de-automize their regular movement, under both single and dual-task (walking + serial sevens) conditions, both before and after 30-min of neurofeedback training. Three different types of neurofeedback were prescribed across three laboratory visits. We hypothesised that training to decrease central EEG alpha-power at scalp sites above the supplementary motor area would facilitate performance compared to opposite (increase central EEG alpha-power) or sham neurofeedback training. Results revealed a pre-test to post-test improvement in performance on the single-task and on both aspects of the dual-task when participants were trained to decrease central EEG alpha-power. There were no benefits of opposite or sham neurofeedback training. Mediation analyses revealed that the improvement in dual-task motor performance was mediated by the improvement in cognitive performance. This suggests that the neurofeedback protocol was beneficial because it helped to reduce conscious control of the motor task. The findings could have important implications for rehabilitation and high-performance (e.g., elite sport) domains; neurofeedback could be prescribed to help alleviate the problems that can arise when individuals exert conscious motor control.

**Electronic supplementary material:**

The online version of this article (10.1007/s00221-020-05935-3) contains supplementary material, which is available to authorized users.

## Introduction

The control of human movements is thought to automize with repetition, promoting consistent and accurate execution at reduced cognitive cost (Fitts and Posner 1967). An important benefit of movement automatization is reduced susceptibility to dual-task interference. For example, healthy adults can perform walking, an everyday movement skill that is established and automatized during early childhood, while concurrently engaging with a range of other motor (e.g., carrying a tray of water) and cognitive (e.g., holding a conversation) tasks (Abbruzzese et al. 2014). However, sometimes movement automaticity degrades, and individuals become inclined to consciously control actions, prompting regression on the skill acquisition continuum towards a more primitive state (Clark 2015; Masters and Maxwell 2008). In their de-automatized states, movements demand more attentional resources, and dual-task situations like the example provided above can result in overloading, an increased likelihood of task prioritization, performance degradation and an elevated risk of falls (Denneman et al. 2018; Kal et al. 2013; Paul et al. 2005). In this experiment, we sought to examine the effects of dual-tasking on de-automized movement. The main objective was to evaluate the utility of electroencephalographic (EEG) neurofeedback—a form of brain training—as an intervention to restore movement automaticity. If effective, neurofeedback training could be prescribed as a non-pharmacological treatment to aid motor performance across any domain where steep learning curves and automatic movements are desired (e.g., movement rehabilitation, high performance sport).

## Movement (de)automization

Brain imaging studies have revealed that walking in healthy adults is characterised by activation of the supplementary motor area of the brain (e.g., Hanakawa et al. 1999). Phasic cues from the basal ganglia are said to activate the supplementary motor area prior to each stepping movement (Morris et al. 1996), and this activation supports automated step initiation and anticipatory postural control to ensure balance regulation during gait (Takakusaki 2013). The idea that the automated control of movements can, on occasion, be undone, is best described by Masters and Maxwell’s (2008) reinvestment theory. In brief, the theory argues that contingencies such as movement errors or increases in anxiety can induce conscious motor processing, often as a well-intentioned coping strategy. However, such lapses into conscious control have adverse effects on the performance of well-learned movements such as walking, because conscious control is more effortful (e.g., fatiguing), slower (e.g., to adjust movements, such as correcting imbalance) and more subject to disruption and failure than autonomous control (Uiga et al. 2020).

One contingency that can promote conscious motor control and impair motor performance is any constraint that forces modification to normal automized movement patterns. For example, Beilock and Carr (2001) demonstrated that when expert golfers hit shots with a regular putter, they had little recollection of their thought processes during each putt—an effect they termed “expertise-induced amnesia”. However, when asked to hit shots with a modified S shaped putter, the experts were able to provide much more detailed recollections of their thoughts during putts, and their performance was impaired. The results were interpreted to indicate that the task constraints induced by the unfamiliar putter forced experts to revert to a more conscious form of processing, de-automizing their normal technique.

Other contingencies that can promote reinvestment include injury or disease. For example, Masters, Pall, MacMahon and Eves (2007) demonstrated that the self-reported propensity to consciously monitor and control movements was higher among people with Parkinson’s disease compared to age matched controls. They also reported that propensity for conscious control increases with Parkinson’s disease duration. This can be attributed to the progressive decay of the key basal ganglia to supplementary motor area brain circuits that are associated with automated motor control (Morris et al. 1996). For example, many people with Parkinson’s display hypoactivation of the supplementary motor area (Hanakawa et al. 1999), and this is associated with gait disturbance indicative of de-automization (Iseki et al. 2010).

To circumvent the difficulties posed by this neurodegeneration, neurophysiological studies have revealed that people with Parkinson’s (and others who attempt to take conscious control of gait) seem to control their movements via increased activation of their frontal cortex (e.g., Maidan et al. 2016), a part of the brain deputed to conscious executive functions and goal-driven processing (Ridderinkhof et al. 2004). This compensation strategy allows mobility to be maintained (Maidan et al. 2019). However, when controlled in this way, walking is characterised by postural instability, slowed gait and a greater risk of falls (Maidan et al. 2016). As the performance of some cognitive operations (e.g., mental arithmetic) is also associated with supplementary motor area activity (Hanakawa et al. 2002), the shift away from the supplementary motor area and towards a more frontal activation during gait also increases susceptibility to dual-task interference. For example, Denneman et al. (2018) demonstrated that a higher propensity for conscious motor control was associated with greater dual-task costs, calculated as the magnitude of impairment in both motor and cognitive performance when a walking and a tone-counting cognitive task were performed concurrently compared to when they were performed alone.

## How to prevent de-automization? neurofeedback training

Considering the problems associated with the de-automization of fundamental movements (e.g., gait), there is a pressing need for interventions that can help people maintain automaticity of movements. One approach that has received little attention in movement rehabilitation to date, but has the potential to directly target dysfunctional patterns of cortical activity and/or encourage the (re)activation of automaticity-related brain circuits, is EEG neurofeedback training. In brief, neurofeedback training involves recording and displaying an individual’s brain activity in real time, while encouraging them to develop strategies to control their brain activity levels. For example, computer software can be programmed to reward a participant by displaying a positive image or emitting a pleasant sound whenever a desired pattern of activation is achieved.

Using a form of auditory neurofeedback where the onset of a tone was used to reward participants for increasing EEG power in the sensorimotor rhythm frequency band (SMR, around 12–15 Hz), Cheng et al. (2015) examined the effects of neurofeedback training in sport. Specifically, they recorded sensorimotor rhythm power at the Cz electrode, which roughly overlies the supplementary motor area (Gerloff et al. 1997; Goel et al. 2019). It was reasoned that augmented SMR power at Cz reduces cognitive interference with movement processing and thereby encourages relatively automatic motor control. Eight experienced golfers were instructed to only hit putts when the tone sounded. Over 4–6 h of training, the participants receiving neurofeedback improved to a greater extent than a control group.

Similarly, in a rehabilitation setting, Fumuro et al. (2013) used visual neurofeedback to encourage 10 people with Parkinson’s disease to increase the negativity of EEG slow cortical potentials recorded at Cz. They selected this type of training because the negative slow cortical potential recorded at the Cz site immediately before movement (better known as the bereitschaftspotential or the readiness potential), has been suggested to reflect supplementary motor area activity, with more negativity indicative of increased supplementary motor area activation (Deecke and Kornhuber 1978; Fumuro et al. 2013). The readiness potential was recorded in the moments preceding each of 100 button press trials completed before and after participants undertook between 104 and 260 (depending on fatigue) 10 s neurofeedback trials where their goal was to control their slow cortical potential (displayed on the screen) so it exceeded a target threshold (also displayed on the screen). A neurofeedback trial was deemed successful if the slow potential amplitude produced by the participant exceeded the target level and remained there for at least 2 s of the final 4 s of each trial. Participants were classified as good or poor performers based on the number of successful trials during their neurofeedback training, and analyses revealed that the good performers displayed a significant increase in the negative amplitude of their readiness potential from the pre- to the post-neurofeedback training button press task. This was interpreted to indicate that individuals are able to increase the negativity of their readiness potential and thereby increase activation of their supplementary motor area before voluntary movement. However, the study did not assess the effects of neurofeedback on motor performance.

Finally, in a more recent experiment, Hindle et al.(2020) utilized auditory neurofeedback to encourage 16 people with Parkinson’s disease to decrease EEG alpha power (around 8–12 Hz) at the central electrodes overlying the supplementary motor area prior to initiating a precision grip force task designed to test their fine motor control. EEG alpha power has an inhibitory function, with increases in alpha power said to inhibit brain areas, and decreases in alpha power said to increase cortical activation (Klimesch et al. 2007). Accordingly, the neurofeedback was designed to increase activation at sites overlying the supplementary motor area in an attempt to encourage activity of brain circuits linked to autonomous motor control.^1^ Results indicated that 3 hours of neurofeedback training was associated with a significant decrease in the time taken to initiate fine motor tasks, thereby implying that neurofeedback encouraged faster motor planning, as would be expected from a more autonomous form of control. However, the report of this experiment has not undergone full peer review and it is only published in summary form at present.

While these studies provide encouraging evidence to support the use of neurofeedback as a means of modifying cortical activity and/or facilitating motor performance, they provide only speculative evidence that increased automaticity of motor control was the mechanism underpinning the neurofeedback benefits. They also contained small samples and were restricted to discrete and precision-based motor tasks. Accordingly, further research is warranted.

## The present experiment

In this experiment, we sought to address the limitations of previous neurofeedback research by examining the effects of neurofeedback training on a whole-body fundamental motor task (i.e., walking) that participants performed while wearing a leg brace to lock the knee, thereby establishing a constraint to de-automize gait (cf. Beilock and Carr 2001). We also implemented a dual-task condition to examine the extent to which any benefits of neurofeedback training on motor performance can be attributed to a relative restoration of movement automaticity. Our design includes a neurofeedback training condition designed to decrease central EEG alpha power at scalp sites above the supplementary motor area, based on the tentative suggestion that this may permeate the supplementary motor area and increase the activation of automaticity-related locomotor brain circuits (cf. Goel et al. 2019; Knaepen et al. 2015). We compare this to two control conditions: an opposite neurofeedback condition (i.e., increase alpha power at Cz); and a sham neurofeedback condition (i.e., fake neurofeedback training). Such control conditions are rarely adopted but are strongly recommended for neurofeedback studies to control for possible placebo effects (sham control) and to allow directional attributions (opposite neurofeedback control) (Thibault et al. 2016). We hypothesized a condition × test interaction effect, where motor performance (walking) would improve from pre- to post-neurofeedback training to a greater extent in the decrease alpha power training condition compared to the two control conditions. We also predicted that this selective improvement in motor performance for the decrease alpha power training condition would be accompanied by a pre- to post-neurofeedback training improvement in cognitive dual-task performance. Finally, we predicted that the pre-test to post-test improvement in dual-task walking performance during the decrease alpha power condition would be mediated by the pre-test to post-test improvement in cognitive performance. These latter two findings would provide novel evidence that the performance benefits of the neurofeedback intervention can be attributed to neurofeedback helping to restore movement automaticity, and thereby freeing up frontal resources to aid dual-task processing.

## Methods

### Participants

Twenty-five participants (Females = 16, *M*_age_ = 23.5, *SD* = 1.37, Males = 9, *M*_age_ = 23.67, *SD* = 1.00 years) volunteered to take part in the experiment. We recruited participants via advertisement posters. All participants reported being free from illness and injury at the time of the experiment. We obtained informed consent from all participants. The experiment was approved by the University research ethics committee.

G*Power 3.1 power calculation software (Faul et al. 2013) indicated that by adopting an alpha of 0.05 and a sample size of 25, the experiment was powered at 0.80 to detect within-participant differences for effect sizes exceeding *f* = 0.26 (i.e., medium-size effects) by repeated-measures analysis of variance (ANOVA; Cohen 1992). In a previous study of the effects of neurofeedback training on motor performance, Cheng et al. (2015) reported a significant and large within-participant effect (*η*_p_^2^ = 0.63; performance improvement from pre- to post-training). Accordingly, if similar effects were to emerge, our sample was adequately powered to detect them.

### Design

**Fig. 1:**
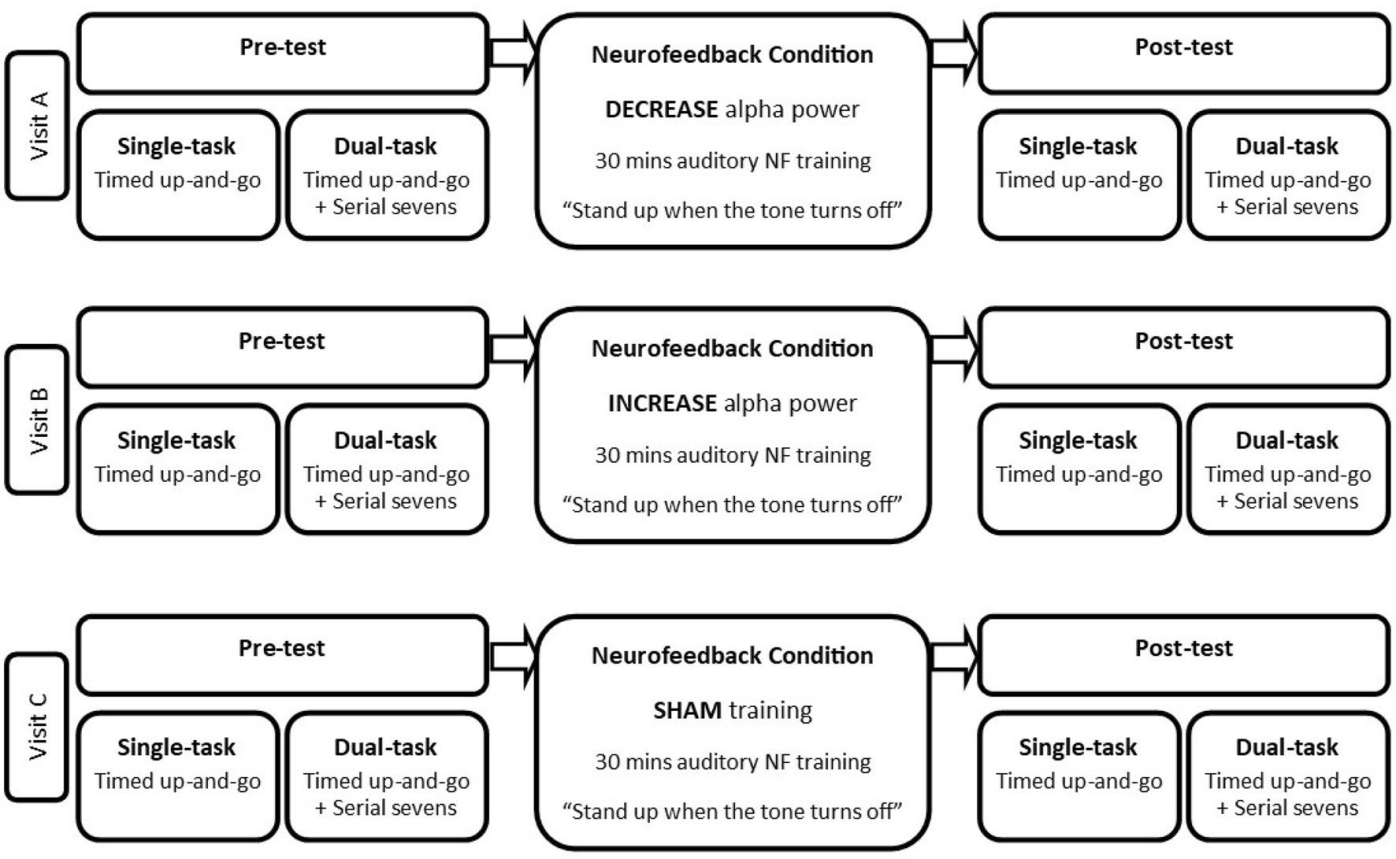
Schematic of experiment design. All participants made three laboratory visits on separate days (Visit A, Visit B, Visit C). During each visit they completed a single-task walking assessment, and a dual-task walking assessment both before (i.e., pre-test) and after (i.e., post-test) 30 min of neurofeedback. The type of neurofeedback differed on each visit (i.e., 3 neurofeedback conditions: decrease alpha, increase alpha, sham). The order of Visits A, B and C were counterbalanced across participants. This made for a fully within-subject 3 Condition (decrease alpha; increase alpha; sham) × 2 Test (pre-test; post-test) × 2 Task (single-task; dual-task) design

We adopted a within-participant design with three primary factors: Task, Test, and Condition. The Task factor had two levels: single-task and dual-task. All participants completed a timed up-and-go walking assessment on its own (i.e., single-task), and while concurrently performing a serial-sevens cognitive task (i.e., dual-task). The Test factor also had two levels: pre-test and post-test. All participants completed their walking tasks both before (i.e., pre-test) and after (i.e., post-test) receiving neurofeedback. The Condition factor had three levels: decrease alpha power, increase alpha power, and sham. Each condition comprised 30-min of neurofeedback training. In the decrease alpha power neurofeedback condition, participants received neurofeedback training that encouraged them to decrease their central midline alpha power. In the increase alpha power neurofeedback condition participants received neurofeedback training that encouraged them to increase their central midline alpha power. In the sham neurofeedback condition participants received non-contingent (i.e., fake) neurofeedback training. All participants made three laboratory visits on separate days to complete all three neurofeedback conditions. This ensured a fully within-participant 3 Condition (decrease alpha; increase alpha; sham) × 2 Test (pre-test; post-test) × 2 Task (single-task; dual-task) design. A schematic of the design is provided in Fig. 1, and a more detailed description of the Task and Condition factors are respectfully provided in the “Task” and in the “Neurofeedback training protocol” sections below.

### Task

#### Single-task: timed “Up and Go” test

The Timed “Up and Go” test (TUG), is an established clinical tool utilized to assess instability, postural control and lower limb functional mobility (Vance et al. 2015). It requires the execution of everyday movements (i.e., standing, walking, turning) therefore, providing an efficacious means of predicting the risk of falls and identifying individuals with impaired motor function (Brustio et al. 2017; Nocera et al. 2013). The TUG test measures the total amount of time required to rise from a seated chair position (seat height 45 cm), walk a distance of 3 m, turn around a cone, walk back to the chair and sit back down. Healthy adults typically complete the task in 8–10 s; times exceeding 10 s normally indicate reduced physical capacity and/or balance and mobility problems, with a positive correlation between TUG and risk of falls (Bohannon 2006; Kear et al. 2017).

#### Dual-task: timed “Up and Go” test + serial sevens

In the dual-task condition participants completed the TUG while simultaneously performing a serial sevens cognitive task. Specifically, we instructed participants to serially subtract by sevens from a prescribed three-digit number (ranged from 100–300) and recite out loud the calculations while performing the TUG test. The serial sevens task loads working memory and increases the demand for attentional resources and is regularly used alongside walking to establish a genuine motor and cognitive dual-task (Montero-Odasso et al. 2012).

### Neurofeedback training protocol

Participants received a 30-min session of neurofeedback training during each of their three laboratory visits. Cortical activity was recorded during the increase alpha and the decrease alpha neurofeedback conditions from the central midline of the scalp (i.e., Cz electrode site; Jasper 1958) which roughly overlies the supplementary motor area (Gerloff et al. 1997), using an active electrode connected to a wireless 4-channel neurofeedback system (Brainquiry PET-4, Nijmegen, Netherlands). Additionally, an active electrode was placed over the orbicularis oculi muscle of the right eye to remove eyeblink artefacts, with reference and ground electrodes attached to the right and left mastoids (Ring et al. 2015). We focused our feedback at the Cz site because the supplementary motor area supports movement planning and posture preparation to ensure balance regulation during gait (Takakusaki 2013), and because supplementary motor area dysfunction is associated with gait disturbance (Iseki et al. 2010). In tandem with cortical recordings, a computer running Bioexplorer software (Cyberevolution) used a 6th order Butterworth infinite impulse response 8–12 Hz bandpass filter to extract alpha power (8–12 Hz) from the EEG signal and fed this back to the participants in the form of an auditory tone (Ring et al. 2015). Importantly, the tone was programmed to vary in pitch based on the level of alpha power and silence completely when alpha power was decreased (during the decrease alpha condition) or increased (during the increase alpha condition) by 30%, relative to each participant’s individual baseline. As alpha power is inversely related with cortical activity, the decrease alpha condition encouraged increased activation at the Cz site above the supplementary motor area, which is characteristic of relatively autonomous locomotion (Morris et al. 1996), and the increase alpha condition encouraged decreased activation above the supplementary motor area, which is characteristic of de-automized locomotion and gait disturbance (Iseki et al. 2010). In addition to changing alpha power by 30%, the system also required < 10 µV of 50 Hz activity in the signal (i.e., low impedance) and the absence of eye-blinks, as detected by the electrode paced adjacent to the right eye (eye-blinks were detected as > 75 µV of 1–7 Hz activity at the eye-electrode), for the tone to silence. These control features helped ensure the signal was being regulated by cognitive processes and was not contaminated by electrical, muscular or eye-blink artefacts (Ring et al. 2015).

The auditory neurofeedback training was delivered to participants over ten 3-min blocks, each separated by a 1-min break. Participants were seated in the chair used for the TUG. Each time the thresholds described above were met, the auditory tone was set to silence for 1.5 s and participants were instructed to stand up from the chair. This instruction was designed to help participants associate relative increased or decreased central midline activation (in the decrease alpha, and increase alpha conditions, respectively) with the onset of movement.

During the sham feedback condition, the procedure was identical except cortical activity was not recorded^2^ and the tone that participants heard was not based on their brain activity. Instead, participants were played a recording of a tone from either their previous visit or from a matched participant taken during one of the other neurofeedback conditions. Accordingly, unbeknownst to the participant, they received no systematic encouragement to change their central midline activation during the sham condition.

### Measures

#### Walking performance

Our primary outcome measure was TUG completion time in the single and dual-task conditions and across the pre-test and post-test phases. This is a well-established performance measure in the gait and movement rehabilitation literature (Zirek et al. 2018). Time to completion scores were obtained by recording each walking trial using a video camera (Apple Ipad) and analysing the time-stamped footage.

#### Cognitive performance

A secondary outcome measure was cognitive task performance. We calculated percentage accuracy of serial seven responses provided during each trial by recording the number of responses and the number of errors (Tamura et al. 2018).

#### Cortical activity

EEG activity was recorded during the increase alpha and the decrease alpha neurofeedback sessions from the Cz site on the scalp (Jasper 1958), using an active electrode connected to a DC amplifier (Brainquiry PET-4), with reference and ground electrodes attached to the right and left mastoids, respectively. Recording sites were cleaned, abraded, and conductive gel (Signagel, Parker) was applied to ensure that electrode impedances were below 10 kΩ. The signals were digitized at 24-bit resolution (Brainquiry) and transmitted via Bluetooth at a sampling rate of 200 Hz to a computer running Bioexplorer (Cyberevolution) software. We employed Butterworth infinite impulse response (6th order) bandpass filters at 8–12 Hz (alpha power), 4–8 Hz (theta power) and 13–30 Hz (beta power) to extract EEG data from each three-minute recording. EEG alpha power was extracted to provide an indication of how the neurofeedback interventions modulated EEG alpha power. Power from the neighbouring theta and beta power bands was extracted to examine whether the effects of the neurofeedback manipulation were localised to the alpha band. The EEG recording system was only switched on during the neurofeedback phase of the experiment. Cortical activity was not recorded during the pre-test and post-test phases of the experiment due to short recording epochs, low number of trials, and risk of the wireless signals dropping out as participants moved away from the data-receiving computer during TUG trials.

### Procedure

Participants attended three separate 75-min testing sessions (increase alpha, decrease alpha, sham) with each visit separated by a minimum of 24 h and a maximum of 7 days (*M* inter-session-interval = 3.61 ± 1.22 days) to reduce the potential for any carry over effects. The order of conditions was counterbalanced across participants. At the onset of the first laboratory visit participants were briefed and provided informed consent. All visits then followed the same identical structure. First, participants were seated and fitted with the neurofeedback system. We prepared the skin by lightly abrading over the mastoids and the right orbicularis oculi muscle with exfoliating paste, and with a blunt needle at the scalp site (Cz). The sites were then cleaned with an alcohol wipe, conductive gel was applied, and disposable spot electrodes (BlueSensor, Ambu) were placed and secured using tape and a lycra cap. The EEG amplifier was attached by an elastic and Velcro strap to the participant’s right arm.

**Fig. 2:**
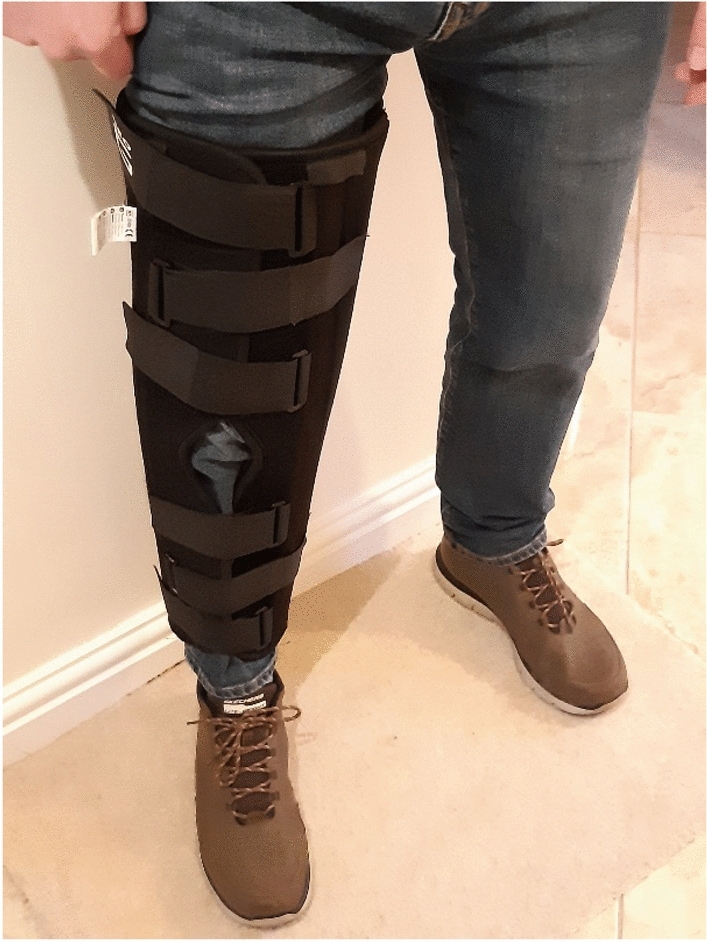
Image depicting the wrap-around aluminium splinted leg brace that was used to immobilise the knee joint and encourage de-automization of the regular gait of our healthy adult participants

After instrumentation, we provided instructions about the TUG test and serial sevens dual-task, and the TUG was demonstrated by the experimenter. Next, the participant completed one TUG familiarisation trial without the leg brace, and we recorded this baseline TUG score. On completion of the familiarisation trial, participants were fitted with a 60 cm wrap around aluminium splint leg brace (Knee Immobiliser, NeoG, Harrogate, United Kingdom—Fig. 2) to immobilise the knee joint of their dominant leg. This feature was designed to disrupt the autonomous control of gait and thereby induce a more conscious form of movement control (Beilock and Carr 2001). De-automizing regular gait was necessary to establish the efficacy of neurofeedback at re-automizing movements, and thereby examine the primary aim of this experiment.

Participants then began the pre-test phase of the experiment, comprising three trials of the single-task and three trials of the dual-task (see task-section above). The order in which they completed the single-task and the dual-task conditions was counterbalanced across participants. There was a 30 s break in between each trial, and a 1-min break in-between the single-task and dual-task conditions.

After the pre-test, the EEG amplifier was switched on and participants were prepared for the neurofeedback phase. We first assessed their baseline alpha power by asking them to fixate on a cross taped to the wall at eye level, for a period of five seconds. During this time, Cz alpha power was monitored. This process was repeated five times and the average was used as their baseline Cz alpha power. Having established individual baselines, the experimenter manually set the threshold for silencing the neurofeedback tone in the neurofeedback software. In the increase alpha condition, the threshold was set as baseline + 30%. In decrease alpha condition, the threshold was set as baseline -30%. In sham condition, the experimenter pretended to enter a threshold in the computer, but no real threshold was set. Participants were then supplied with the following instructions:

“In the next phase of the experiment the computer will play an auditory tone that is based on your real time brain activity. When you reach the target level of brain activity, the tone will turn off and that is your cue to stand up. The neurofeedback training session will last 30 min, in the form of ten 3-min blocks, interspersed with short breaks. Try to recognize how to control the tone with your thoughts. It may be difficult at times, but it should become easier with practice. The goal during each 3-min block is to silence the tone and stand up out of the chair as many times as you can.”

Participants completed the ten 3-min blocks of neurofeedback training, with a 1-min break between each block. On completion of the neurofeedback training, the EEG amplifier was switched off and participants entered the post-test phase, which comprised three trials of the single-task and three trials of the dual-task, in the absence of the auditory tone. This was identical to the pre-test. At the end of each session the leg-brace and neurofeedback hardware were removed, and the participant was thanked and reminded of the time and date of their next visit. At the end of the third visit participants were thanked for a final time and invited to contact the experimenter for the results of the experiment at the end of the data collection period.

### Statistical analyses

#### Cortical activity

We analysed cortical activity during the decrease alpha power and during the increase alpha power neurofeedback training sessions. Separate 2 Condition (decrease alpha, increase alpha) × 10 Block (each of the 3 min neurofeedback sessions) ANOVAs were performed for the alpha, theta, and beta power bands. We expected a Condition × Block interaction to emerge for the alpha band, characterised by significantly greater alpha power in the increase alpha condition than in the decrease alpha condition during the final block of neurofeedback training. We expected no significant effects to emerge in the theta or beta power bands. Significant ANOVA effects were probed by polynomial trend analyses and paired samples t-tests. EEG data were missing for three participants in the increase alpha and one participant in the decrease alpha conditions due to software error. Missing data are reflected in the reported degrees of freedom.

#### Main analyses

Our primary hypotheses concern walking performance and cognitive performance (i.e., serial sevens response accuracy), so these variables were the focus of our main analyses. We analysed walking performance using a 3 Condition (decrease alpha, increase alpha, sham) × 2 Test (pre-test, post-test) × 2 Task (single-task, dual-task) repeated measures ANOVA. We analysed cognitive performance using a 3 Condition (decrease alpha, increase alpha, sham) × 2 Test (pre-test, post-test) ANOVA. Significant effects were probed by paired-samples t-tests.

Finally, to test our hypothesis that increased automaticity would be responsible for any pre-test to post-test improvement in dual-task walking performance during the decrease alpha power neurofeedback condition, we performed within-participant mediation analyses (Montoya and Hayes 2017). We set pre-test and post-test dual-task walking performance scores from the decrease alpha power condition as the dependent variables and pre-test and post-test serial-sevens response accuracy scores from the decrease alpha power condition as the potential mediator variables using MEMORE for SPSS (MEdiation and MOderation analysis for REpeated measure designs; Montoya and Hayes 2017). The mediation effect (B), standard error (BootSE) and 95% CI (low and high) were reported (Montoya and Hayes 2017).

#### Control analyses

In addition to our main analyses, we also conducted some control analyses to test some assumptions of our experiment and provide further insight into the data. We tested our assumption that walking with a leg brace would slow gait akin to what happens when individuals revert from autonomous to conscious motor control (Beilock and Carr 2001). We tested the assumption of neurofeedback experiments that participants progressively gain more control over their brain activity as their training sessions progress by analysing the number of times that participants stood up during neurofeedback training. Finally, we analysed number of serial-sevens responses to screen for possible speed-accuracy trade-offs in cognitive task performance. The results of these analyses are presented in the Supplementary Material. In brief, both our assumptions were confirmed, and there was no evidence of speed accuracy trade-offs.

## Results

### Cortical activity

**Fig. 3:**
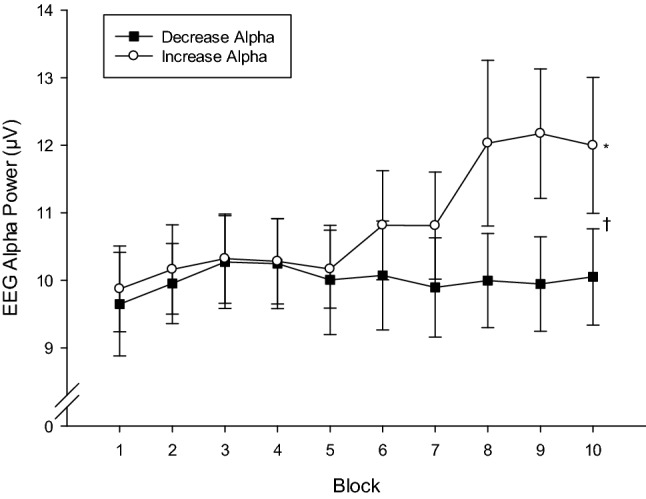
Condition × Block interaction effect on EEG Alpha power during neurofeedback training. *Indicates significant increase in alpha power across blocks during the increase alpha power condition (*p*  < . 01). †Indicates significant between-condition difference in alpha power during blocks 9 and 10 (*p* < .01). Error bars depict standard error of the means

A 2 Condition × 10 Block ANOVA performed on EEG alpha power during the neurofeedback phase of the experiment revealed a significant Condition × Block interaction, *F*(9,12) = 3.17, *p* < 0.05, *η*_p_^2^ = 0.70. EEG alpha power was always higher during the increase alpha condition, and polynomial trend analyses confirmed a significant linear increase in alpha power across blocks, *F*(1,21) = 8.38, *p* < 0.01, *η*_p_^2^ = 0.29, while alpha power in the decrease alpha condition remained relatively low and stable throughout (Fig. 3). Paired samples *t*-tests confirmed that EEG alpha power was significantly higher in the increase alpha power condition than in the decrease alpha power condition during the final block of neurofeedback training, *t*(20) = 3.08, *p* < 0.01. There were no Condition × Block interactions in the theta or beta power bands, *F*’s(9,12) = 0.70–1.77, *p*’s = 0.18–0.70, *η*_p_^2^′s = 0.34–0.57, and there were no Condition or Block main effects in any of the bands, *F*’s = 0.43–2.37, *p*’s = 0.14–0.78, *η*_p_^2^′s = 0.02–0.38. These analyses confirm that the neurofeedback interventions had a selective effect on EEG alpha power and established distinct and a relatively higher level of EEG alpha power at the end of the increase alpha power neurofeedback training, and a lower level of EEG alpha power at the end of the decrease alpha power neurofeedback training.

### Main analyses

Having provided some evidence of the effectiveness of the neurofeedback interventions in establishing distinct levels of EEG alpha power at the end of the neurofeedback training, we preceded with analyses of our primary hypotheses concerning the effects of neurofeedback condition on behavior during the pre- and post-tests.

### Walking performance

**Fig. 4:**
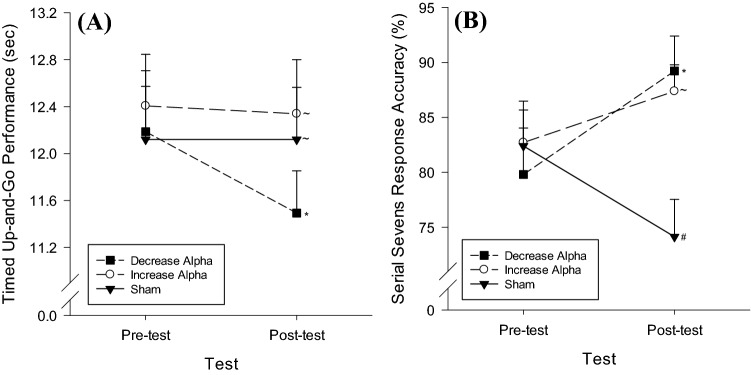
**a** Condition × Test interaction effect of neurofeedback on walking performance. **b** Condition × Test interaction effect of neurofeedback on cognitive performance. *Indicates significant improvement from pre-test to post-test (*p* < 0.02). ~ Indicates no significant change from pre-test to post-test. # Indicates significant decline from pre-test to post-test (*p* < 0.02). Error bars depict standard error of the means

A 3 Condition (decrease alpha, increase alpha, sham) × 2 Test (pre-test, post-test) × 2 Task (single-task, dual-task) ANOVA conducted on mean TUG performance scores revealed main effects for Test *F*(1,24) = 6.49, *p* < 0.05, *η*_p_^2^ = 0.21 and Task *F*(1, 24) = 34.82, *p* < 0.05, *η*_p_^2^ = 0.59. Paired samples t-tests revealed TUG performance improved from pre-test (*M* = 12.24, SD = 2.25 s) to post-test (*M* = 11.98, SD = 1.98 s). Additionally, TUG trials were performed faster in the single-task (*M* = 10.77, SD = 1.61 s) than in the dual-task (*M* = 13.45, SD = 2.97 s) conditions. Importantly, there was also a significant Condition × Test interaction, *F*(2,23) = 3.78, *p* < 0.05, *η*_p_^2^ = 0.25 (Fig. 4a). Post-hoc *t*-tests indicated that the performance in both the single-task and the dual-task conditions improved from pre-test to post-test during the decrease alpha neurofeedback condition, *t*’s(24) = 2.52–3.02, *p*’s < 0.02, while there were no pre-test to post-test changes in single or dual-task performance during the increase alpha or the sham conditions, *t*’s(24) =  − 0.05–0.852, *p*’s = 0.40–0.96. No other main or interaction effects were significant.

### Cognitive performance

A 3 Condition (decrease alpha, increase alpha, sham) × 2 Test (pre-test, post-test) ANOVA employed to analyse serial sevens response accuracy during the dual-task phase of the experiment revealed no main effects for Condition, *F*(2,23) = 2.91, *p* = 0.08, *η*_p_^2^ = 0.20 or Test, *F*(1,24) = 0.61, *p* = 0.44, *η*_p_^2^ = 0.03. Importantly, there was a significant Condition × Test interaction, *F*(2,23) = 11.31, *p* < 0.05, *η*_p_^2^ = 0.50 (Fig. 4b). Post hoc paired sample *t*-tests indicated that response accuracy significantly increased from pre-test to post-test during the decrease alpha neurofeedback condition, *t*(24) = − 2.68, *p* < 0.02. Response accuracy significantly decreased from pre-test to post-test during the sham neurofeedback condition, *t*(24) = 2.67, *p* < 0.02. Finally, response accuracy did not change from pre-test to post-test during the increase alpha neurofeedback condition, *t*(24) = − 1.00, *p* = 0.33.

### Mediation analyses

Mediation analyses were used to examine whether within-participant changes in pre-test to post-test serial sevens performance mediated changes pre-test to post-test walking performance in the decrease alpha power neurofeedback condition. They revealed a significant and positive indirect effect of test (pre-test, post-test) on dual-task walking performance via serial sevens accuracy (indirect effect = 0.24, BootSE = 0.18, 95% confidence intervals [0.00–0.69]). This indicates that 240 ms of the average 880 ms pre-test to post-test improvement in dual-task TUG score observed in the decrease alpha power neurofeedback condition can be attributed to the pre-test to post-test improvement in cognitive performance.

## Discussion

This experiment evaluated whether neurofeedback training can benefit motor performance by encouraging a shift towards more automatic control of movements. Specifically, we used a leg brace to disrupt and de-automatize the control of walking, and then we applied neurofeedback to examine its effects on both single and dual-task walking performance. We hypothesized that walking performance would improve from pre- to post-neurofeedback training to a greater extent in the decrease alpha power training condition compared to the two control conditions. This is because training to decrease central alpha power may encourage activation over the supplementary motor area, thereby replicating activity patterns that characterise relatively autonomous locomotion (Morris et al. 1996). Our hypothesis was supported. Specifically, walking performance measured by TUG scores improved significantly from pre-test to post-test on both single-task and dual-task trials during the decrease alpha power condition. Overall, the decrease alpha power neurofeedback training was associated with a 6% improvement in TUG score from pre-test to post-test, while no significant improvements were observed in either of the control conditions. This finding complements existing evidence arguing a similar beneficial effect of decrease central alpha power neurofeedback training on the time required to initiate discrete motor tasks (Hindle et al. 2020). Importantly, it provides new evidence that EEG-based neurofeedback training designed to increase central midline activation can have a beneficial effect on the performance of whole-body fundamental movements.

To further extend previous work, we tested whether the benefits of decrease central alpha power neurofeedback training on motor performance can be attributed to the adoption of a more automatic form of motor control. First, we predicted that the decreased alpha power neurofeedback training condition would be associated with a pre- to post-neurofeedback training improvement in cognitive dual-task performance. This hypothesis was supported; performance on the cognitive dual-task significantly improved by 9% from pre-test to post-test during this neurofeedback condition. In the other two neurofeedback conditions, cognitive performance was either unchanged (i.e., non-significant 4% improvement in the increase alpha power condition) or was worse (i.e., significant 8% decline in the sham condition) after the neurofeedback training. Therefore, the decrease central alpha power neurofeedback was the only form of neurofeedback associated with improvements in both cognitive and motor performance. Since the simultaneous performance of both cognitive and motor tasks can increase attentional loading (Montero-Odasso et al. 2012), dual-task situations pose an elevated risk of attentional overload and performance difficulties for both tasks (Brustio et al. 2017). This risk is reduced when either task is performed in a more automatic fashion, reducing the need for conscious attention-demanding forms of control. It is possible that decreased alpha power neurofeedback training helped to automize the motor task (walking) thereby speeding up gait, while also freeing up attentional resources to permit superior cognitive performance.

To shed more light on this interpretation of our data, we can consult our final hypothesis. Specifically, we predicted that the pre-test to post-test improvement in dual-task walking performance during the decrease alpha power condition would be mediated by the pre-test to post-test improvement in cognitive performance. We reasoned that support for this prediction would provide more compelling evidence than is currently available (e.g., Cheng et al. 2015; Hindle et al. 2020), to show that the benefits of this neurofeedback training can be attributed to increased motor automaticity. This is because our prediction would statistically associate improved performance on the cognitive dual-task (i.e., a finding known to occur with increased motor automaticity—Kal et al. 2013) with the improvement in motor performance. Mediation analyses supported our prediction, evidencing a significant indirect effect of test on dual-task walking performance (i.e., pre-test to post-test TUG improvement) via serial sevens accuracy. To our knowledge, this is the first mediational evidence of causality in the neurofeedback and human movement literature. Taken together, all the findings provide encouraging evidence that neurofeedback training designed to activate the motor circuits that characterise autonomous control of gait may facilitate relatively autonomous walking performance.

## Cortical activity

Analyses of EEG data recorded during the neurofeedback phase of the experiment confirmed that the increase alpha power neurofeedback condition was successful in establishing a relatively high and increasing level of central mid-line alpha power over the course of the neurofeedback training. Meanwhile, the decrease alpha power neurofeedback condition ensured a sustained and relatively supressed level of central midline alpha power throughout training.^3^ Importantly, there were no differential effects of the neurofeedback conditions on power in neighbouring theta and beta power bands, thereby limiting the possibility that any effects of the interventions could have been caused by changes in cognitive states such as fatigue or stress, which have been respectively linked to theta and beta power (Díaz et al. 2019; Wascher et al., 2014). Most importantly, the two conditions produced patterns of alpha power that were significantly different from one another, and were characterised by relatively greater alpha power (tentatively interpreted to reflect less activation of supplementary motor area; increase alpha condition) and relatively less alpha power (tentatively interpreted to reflect more activation of supplementary motor area; decrease alpha condition) by the end of the neurofeedback training in preparation for the TUG post-tests. These findings provide encouraging brain data to support our interpretation of the behavioral differences observed at post-test being as associated with modified patterns of cortical activity induced by the preceding neurofeedback interventions. However, we concede that to conclusively link brain and behavior, we needed to record EEG data during the TUG tasks in the pre- and post-tests during all three experimental conditions (i.e., decrease, increase and sham). Future studies could adopt more TUG trials and obtain event-related recordings to establish how cortical activity during gait changes from pre- to post- neurofeedback training. Such research should also use the during-gait EEG data to perform mediational analyses to formally test the hypothesised link between pre-test to post-test behavioral changes, and between-condition behavioral differences, with the changes in EEG activity induced by various neurofeedback interventions.

## Limitations and future directions

This experiment is not without limitations. First, the only gait parameter we measured was speed. Although changes in gait speed provide a general indication of functional performance, cognitive decline and the risk of falls, there are multiple ways through which changes in speed occur, including changes to cadence and/or stride length variability (Morris et al. 1996). These more fine-grained performance variables could be assessed via motion analysis to provide more detailed insight into how neurofeedback benefits walking performance.

Second, we de-automized movement using an artificial constraint. This de-automization method was effective, as indicated by our control analyses evidencing slower performance when wearing the constraint than when walking normally. However, it is not clear how our results would transfer to patient populations whose movement difficulties are associated with neurodegeneration rather than artificial constraints. A replication of this experiment in a clinical population (e.g., Parkinson’s disease) could shed light on this issue. It is promising to note that preliminary work has provided some evidence of efficacy of neurofeedback training in such populations (e.g., Hindle et al. 2020; Fumuro et al. 2013), suggesting that neurofeedback could be effective even when some degeneration of the supplementary motor brain area is apparent.

Third, we concede that the present experiment provides little insight into the longevity of any neurofeedback effects. It is likely that more than 30-min of neurofeedback would be required for sustained performance benefits, especially in clinical populations. Future studies should investigate multi-day neurofeedback interventions with delayed retention tests to better examine neurofeedback learning and the longevity of any neurofeedback benefits (Ring et al. 2015).

Fourth, future studies would do well to develop neurofeedback interventions based on estimates of the sources (i.e., neural generators) of the signals recorded on the scalp (e.g., Congedo et al. 2004). In the current experiment, we trained participants to increase cortical activation at sites overlaying the supplementary motor area, but we cannot exclude the possibility that we captured some cortical activation from other brain areas as well (Mima and Hallett 1999). Neurofeedback incorporating source localization analyses could target supplementary motor area activation more precisely. To date, very few researchers have taken this source localization approach to neurofeedback and motor performance (for an example see Koberda and Stodolska-Koberda 2014), but with continual improvements in computer modelling and processing speeds, these techniques should be increasingly possible in the future.

Fifth, future studies should ensure that they continue to adopt appropriate control conditions. Here we adopted sham and opposite training control conditions. The results observed during the sham condition provide evidence that the neurofeedback benefits observed during the decrease alpha power condition were genuine rather than being driven by placebo. However, the results observed during the increase alpha power condition were not opposite to the decrease alpha power condition. Since the leg brace de-automized movement in this experiment, training to increase central alpha power (characteristic of de-automized gait—Iseki et al. 2010) may not have been able to further de-automate the skill. It would be interesting to see if increased alpha power training de-automates regular gait without the use of any constraints.

Finally, future studies would do well to compare the current decrease central alpha power neurofeedback protocol to other approaches that are also targeted at re-automizing / preventing the de-automization of movement. For example, increased SMR power neurofeedback (Cheng et al. 2015) or learning via holistic process goals or analogies (e.g., Jie et al. 2016) may be expected to yield similar effects.

## Conclusion

In conclusion, this is the first experiment to evidence that decrease central alpha power neurofeedback training benefits whole body performance by encouraging increased motor automaticity. Our results highlight the potential benefits of neurofeedback training for any motor performance domain where steep learning curves and automatic movements are desired. For instance, neurofeedback could be used to help lessen or alleviate some of the performance problems associated with conscious motor control (Masters and Maxwell 2008). Future studies can build on this initial evidence to examine the effects of our neurofeedback protocol in clinical populations, where there might be considerable potential for neurofeedback as a non-pharmacological adjunct treatment for the motor symptoms of many movement disorders characterised by excess conscious control.

## Electronic supplementary material

Below is the link to the electronic supplementary material.

Supplementary file1 (DOCX 25 kb)

## Data Availability

Data and materials are available upon reasonable request to the corresponding author.

## Footnotes

1 We note that EEG has poor spatial resolution and activity recorded at an electrode overlying the supplementary motor area does not necessarily reflect activation of the supplementary motor area itself. However, studies that recorded EEG during gait and performed source localization analyses have identified the supplementary motor area as one of the generators of scalp recorded electrocortical activity at Cz (Knaepen et al. 2015), and studies applying transcranial magnetic stimulation to create a virtual lesion of the supplementary motor area have revealed that this influences EEG alpha power recorded at Cz and eight other sites that anatomically surround the supplementary motor region (Goel et al. 2019). Accordingly, while linking EEG activity recorded at any scalp site to excitability of a specific brain structure is always somewhat speculative, these findings do provide some tentative evidence of a plausible link between alpha power at Cz and supplementary motor area excitability during gait.

2 While the EEG equipment was attached during the sham condition, we were unable to record cortical activity because the software used to receive EEG signals was set to “playback” rather than “record” mode, to display the non-contingent pre-recorded EEG signals to participants.

3 One might have expected EEG alpha power to decrease across blocks during the decrease alpha power condition; the absence of this effect may be explained by the nature of the EEG recordings. EEG alpha power was averaged over 3-minute blocks. In each block of the decrease alpha power condition participants were training to decrease alpha power and were told to stand up from their chair whenever the neurofeedback tone silenced (i.e., when the required decrease in alpha power had been achieved). Accordingly, each 3-minute recording effectively comprised a series of discrete standing events, preceded by a period of alpha power control (i.e., participants monitoring the neurofeedback tone and using cognitive strategies to decrease alpha), and followed by a few seconds of resetting where participants momentarily disengaged from the tone as they got back into position ready to re-focus and attempt to decrease alpha again. It is possible that alpha power increased substantially as participants reset, relaxed and temporarily relinquished their efforts to control the neurofeedback tone after each standing event, washing out any trend for block by block reductions in alpha power. Unfortunately, our wireless neurofeedback system did not permit timestamp marking of standing events, so we are unable to remove these post-standing epochs from the EEG recordings.
