# Supplementary material for: Electroencephalographic neurofeedback training can decrease conscious motor control and increase single and dual-task psychomotor performance
Source: Exp Brain Res. 2020 Nov 9;239(1):301–13. doi: 10.1007/s00221-020-05935-3 (PMC7884304; doi:10.1007/s00221-020-05935-3)
Supplement: Supplementary file 1 — Supplementary file1 (DOCX 25 kb) [file 221_2020_5935_MOESM1_ESM.docx]

**Supplementary Material**

**Control Analyses**

In addition to the main analyses reported in our manuscript, we performed some control analyses to test some assumptions of our experiment and provide further insight into the data. First, to test the assumption that asking participants to walk with a leg brace would slow their gait, we performed a 3 Condition × 3 Task ANOVA comparing the pre-test TUG scores from the single and dual-task conditions with the TUG score from the familiarisation trial performed before the leg brace was affixed. This 3 Condition × 3 Task (familiarisation without leg brace, pre-test single-task with brace, pre-test dual-task with brace) ANOVA revealed a significant main effect for task, *F*(2,23) = 35.95, *p* <.001, η_p_^2^ = .76. As expected, gait was significantly faster in the familiarisation (no leg brace) trial (TUG *M* = 9.38, *SD*  = 1.70 s) than it was in either of the pre-test (with leg brace) tasks (single-task TUG *M* = 10.86, *SD*  = 1.74 s; dual-task TUG *M* = 13.61, *SD*  = 3.09 s). These analyses indicate that our participants performed to the level expected of individuals with no gait impairment in the familiarisation trial (i.e., TUG < 10 s), and were impaired to a level characteristic of de-automized gait when the brace was worn (i.e., TUG > 10 s) (Bohannon, 2006; Kear, Guck & McGaha, 2017).

A second assumption of neurofeedback experiments is that participants progressively gain more control over their brain activity as their training sessions progress. In our experiment, the number of times that participants stood up per each 3-min block of neurofeedback training reflects the number of times that the tone was silenced, and the prescribed cortical activity was achieved. Accordingly, we subjected the two genuine neurofeedback conditions (i.e., decrease alpha power, increase alpha power) to a 2 Condition × 10 Block (i.e., each of the 3 min neurofeedback sessions) ANOVA, followed by polynomial trend analyses to explore the nature of any change over the 10 time points. This 2 Condition × 10 Block (each of the 3 min neurofeedback sessions) ANOVA revealed no multivariate effects of Condition, Block, or Block × Condition, *F*’s = 0.80 – 3.34, *p* ‘s = .08 - .62, η_p_^2^’s = .12 - .31. However, as the traditional skill acquisition curve is non-linear, we followed these analyses by consulting the polynomial trends. As expected, trend analyses confirmed a significant quadratic trend for Block, *F*(1,24) = 6.16, *p* <.02, η_p_^2^ = .20, characterised by an initial improvement in tone control during the early blocks of each condition, and then a plateau towards the end of training (Supplementary Figure 1).

**Supplementary Figure 1.** Number of times stood up during each neurofeedback block. Error bars depict standard error of the means.

Finally, we performed a 3 Condition × 2 Test ANOVA on number of responses during the serial sevens task. This analysis allowed us to screen for possible speed-accuracy trade-offs in cognitive task performance. The 3 Condition × 2 Test ANOVA revealed no significant effects for Condition, Block, or Block × Condition, *F*’s = 1.23 – 2.96, *p*’s = .07 - .28, η_p_^2^’s = .05 - .20. This indicates that number of serial seven responses per trial (*M* = 3.21, SD = 0.94) was stable across conditions and tests, and the response accuracy results reported in the main analyses above were not contaminated by speed-accuracy trade-offs.
